# HISDAC-US, historical settlement data compilation for the conterminous United States over 200 years

**DOI:** 10.1038/sdata.2018.175

**Published:** 2018-09-04

**Authors:** Stefan Leyk, Johannes H. Uhl

**Affiliations:** 1 Department of Geography, University of Colorado Boulder, 260 UCB, Boulder, CO 80309, USA

**Keywords:** History, Geography, Interdisciplinary studies, Sustainability

## Abstract

Human settlement plays a key role in understanding social processes such as urbanization and interactions between human and environmental systems but not much is known about the landscape evolution before the era of operational remote sensing technology. In this study, housing and property databases are used to create new gridded settlement layers describing human settlement processes at fine spatial and temporal resolution in the conterminous United States between 1810 and 2015. The main products are a raster composite layer representing the year of first settlement, and a raster time series of built-up intensity representing the sum of building areas in a pixel. Several accompanying uncertainty surfaces are provided to ensure the user is informed about inherent spatial, temporal and thematic uncertainty in the data. A validation study using high quality reference data confirms high levels of accuracy of the resulting data products. These settlement data will be of great interest in disciplines in which the long-term evolution of human settlement represents crucial information to explore novel research questions.

## Background & Summary

How and at what rate did key demographic processes such as urbanization or rural-urban transitions evolve at fine granularity and over long time periods? And what can we learn from it for future development of urban systems and landscape fragmentation over large geographic scales? Answering these kinds of questions requires a profound knowledge and understanding of settlement histories, demographic compositions and landscape evolution over large geographic areas, at fine spatial resolution and over extended periods of time. Such knowledge is very limited to date, mainly due to the lack of current data infrastructure that would enable researchers to carry out such retrospective analysis.

Existing historical data are limited to times since medium and fine resolution satellite remote sensing technology became fully operational (i.e., since the 1970s) to produce land cover databases such as the National Land Cover Database (NLCD)^1–3^, population grids such as the Gridded Population of the World (GPW)^4,5^ and its accompanying Global Rural-Urban Mapping Project (GRUMP)^6^, the WorldPop population distribution datasets^7,8^, the Global Urban Footprint (GUF)^9^ and the LandScan population datasets^10,11^ or human settlement layers such as the Global Human Settlement Layer (GHSL)^12,13^. Besides the limited temporal coverage, such data products suffer from low classification accuracy levels in rural and peri-urban settings^14^ thus missing relevant settlement activity. Other potentially useful historical data sources for retrospective national-scale analysis include digitized historical maps. However, maps are only available for certain points in time and contain only specific map layers at a given map scale^15–20^. Furthermore, extracting data from large-volume digital archives of scanned documents remains a computationally challenging task^21^. Historical census data, while becoming increasingly available^22^ (Data Citation 1) are limited in their ability to represent fine-grained human settlement activity, but also in their temporal coverage and resolution.

In this article we describe the creation and properties of novel historical human settlement layers from 1810 to 2015, with a temporal resolution of five years and a spatial resolution of 250 m for most of the conterminous United States. We exploit the potential of large-volume collections of geocoded housing and property-level data: The Zillow Transaction and Assessment Dataset (ZTRAX) is based on existing cadastral data sources and contains more than 374 million data records (https://www.zillow.com/ztrax/). We created two data products. First, we produced a series of multi-temporal raster layers representing the built-up intensity, the sum of gross indoor area of all built structures in a cell at a given year. Second, we built a raster layer that indicates for each raster cell the year of the first settlement. Such unique series of settlement layers have the potential of redefining our understanding of the evolution of human built environments and rural-urban transitional processes that shaped the North American settlement history over two centuries. They will be in high demand by researchers in the social and natural sciences including landscape ecology, demography or urban geography for questions related to resource assessment, natural hazards, vulnerability or land cover change. While the original input data are proprietary records, we established a data use agreement with the owner of the data (the real estate company Zillow) that grants permission to the authors to produce data derivatives that can be provided and disseminated as public data to the research community.

**Figure 1:**
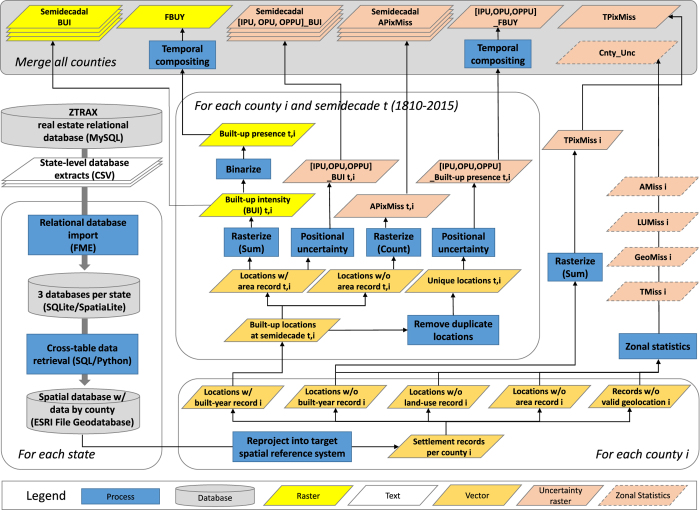
Workflow for the production of the main data products, FBUY and BUI, and the associated uncertainty layers. This figure illustrates the complete workflow including the data processing and integration steps, the creation of the historical settlement layers and the accompanying uncertainty layers.

As can be seen in Fig. 1, this Data Descriptor outlines how we (1) created a spatial database from the raw text-formatted input data, (2) conducted spatio-temporal queries and geoprocessing steps to create the raster layers, and (3) built uncertainty surfaces for informed and critical future data use. The raster layers are consistent with existing global human settlement layers (GHSL) to facilitate data use and integration. We evaluated the data products against a highly accurate validation sample of building footprints with temporal information covering 29 U.S. counties.

## Methods

The owner of the data, Zillow Group, is an online real estate database company that was founded in 2006. The company established several collaborations with academic institutions for scientific use of their home and property database. The historical, fine-grained settlement layers described herein are a first example of a scientific data derivative created from this database. Below, we will describe how we imported the raw ZTRAX data into an efficient database management system, created the historical settlement layers and computed different types of accompanying uncertainty layers.

### Raw data import and spatial database design

The raw ZTRAX data as provided by Zillow consists of almost 2,500 files in Comma Separated Values (CSV) format and has a total volume of 1.4 TB. These files are MySQL database extracts, each representing a table from the original ZTRAX database. The data are delivered as separate databases per state / territory (52 in total). Each state/territory-level database scheme has been split, thematically, into assessment, transaction, and historical assessment data and contains around 50 database tables with a total of approximately 1,000 attributes. Overall, the database tables in the delivered ZTRAX database contain more than 10 billion rows. Based on the delivered ZTRAX metadata we visualized the database scheme using Entity-Relationship diagrams in order to explore the database structure and the relationships between database tables.

In order to ensure efficient and effective use of the data it was crucial to import the raw files into a database system which supports: (i) spatial and aspatial queries, (ii) easy maintenance and further extension once data updates are available, and (iii) portability across systems to facilitate data transfer and sharing between group members. Therefore, we decided to build a system of SQLite/SpatiaLite (https://www.sqlite.org and https://www.gaia-gis.it/fossil/libspatialite/index, respectively) databases, which was preferred over more performant but also less portable products such as PostgreSQL (https://www.postgresql.org/) or MySQL (https://www.mysql.com/) database management systems.

First, we imported the content of the CSV files into 156 SQLite databases (3 per state) using the Feature Manipulation Engine (FME) (https://www.safe.com/), a data processing tool that is able to handle the large volume of the data. The resulting database files have a volume of approximately 1.7 TB in total. Based on the geographic coordinates included as attributes in the database tables, we created SpatiaLite geometries to enable spatial queries in the SQLite databases. Once the import was successful, we compared the metadata provided by Zillow that indicated the number of exported rows for each table to the row counts of our SQLite tables in order to guarantee complete data transfers.

### Creating vector time slices of built-up locations, multi-temporal built-up intensity (BUI) raster layers and a first built-up year (FBUY) layer

The 52 assessment databases contain approximately 150 million records representing cadastral parcels with detailed information on property, land use type, parcel size, built year, information about structures contained in the parcels, and geospatial location, all stored across different (but linkable) database tables. We stored these attributes in geospatial vector tables and then used the data, subsequently, to create multi-temporal settlement layers and accompanying uncertainty surfaces based on geospatial rasterization techniques.

Given the volume of the data it was important to keep computational costs to a minimum and to parallelize data processing where possible. As illustrated in Fig. 1, we first imported the data into state-level SQLite databases, and carried out the subsequent queries and processing steps separately by county. We used the county identifiers that were given as alphanumeric attributes to first extract all records of each county, non-spatially, using pure SQL statements and without applying computationally intensive point-in-polygon queries. Subsequently, we joined selected attributes of interest (e.g., built year, parcel size, building area, etc.) from other database tables and exported these joined tables into an intermediate geospatial vector database to facilitate further geoprocessing (i.e., point geometries in ESRI file geodatabase format). During this step, we excluded data records of land use types that did not indicate the presence of a built structure.

**Figure 2:**
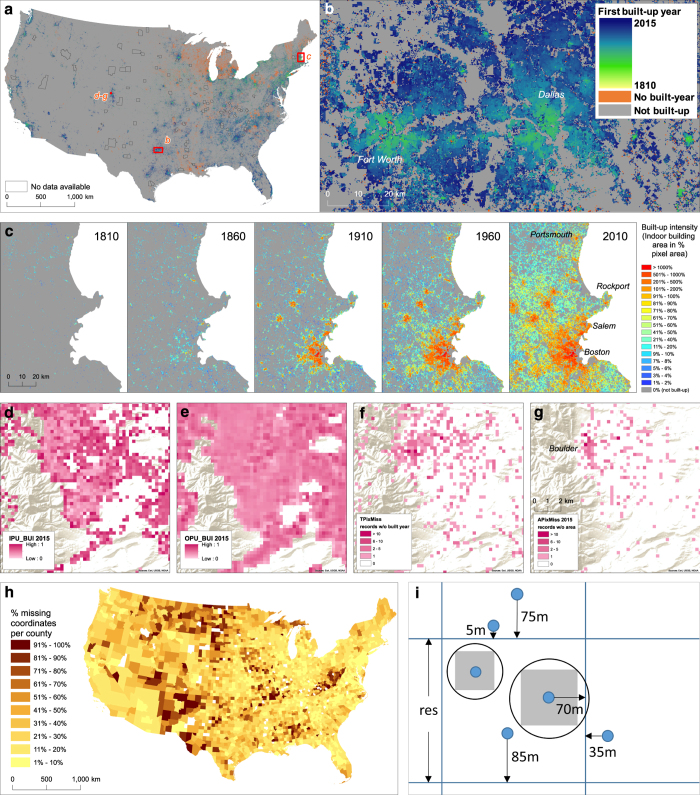
The main settlement data products, BUI and FBUY, their associated uncertainty layers and methodological details for positional uncertainty computation. (**a**) FBUY composite raster layer for the conterminous U.S. with counties where no data is available shown in grey outlines, (**b**) FBUY composite raster layer for greater Dallas-Fort Worth (Texas), (**c**) Selected BUI layers for greater Boston (Massachusetts) for 1810, 1860, 1910, 1960 and 2010, (**d**) IPU_BUI for the year 2015, (**e**) OPU_BUI for the year 2015, (**f**) TPixMiss, (**g**) APixMiss for the year 2015, all (**d**–**g**) for South Boulder (Colorado), (**h**) GeoMiss at county level, (**i**) IPU calculation, and OPU calculation accompanying text descriptions.

Next, we split these point features into five groups according to the availability of relevant attributes needed for rasterization (i.e., built-year, land use type, built-up area, and valid geolocation). We iteratively queried those features that contained temporal information by the built-year attribute (i.e., the year on record at which an existing structure has been built), to generate spatial distributions of existing built-up locations at a given point in time. Starting with the earliest date in each county-level table, we created time slices of point distributions with a built-year equal or prior to the point in time of interest (i.e., temporally cumulative) for every 5 years. In each time slice, we then used all vector features that contained valid gross building area information as input to a rasterization step. This step created gridded cells with a spatial resolution of 250×250 m and a cell value indicating the sum of the gross building area, the registered indoor areas of a structure, from all point features found within the limits of that raster cell. We call this value and hence this raster layer ***built-up intensity (BUI)*** (Figs. 1 and 2c) (Data Citation 2). We did not use the number of features within a cell to assign the raster values because the uncertainty in these counts can become very high and requires further processing. Since these features represent individual properties, a feature could represent a single residential unit such as a single-family home but also a large multi-story building depending on the property ownership situation. The built-up intensity based on gross (indoor) building area represents a more objective reflection of the size of a building, more strongly related to human use and settlement in general. Importantly, we chose the cell size, the spatial reference, as well as the cell alignment to be identical to the GHSL 250 m built-up grid product (projected into Albers equal area conic projection), which facilitates cross-comparison as well as a temporal extension of the settlement layer to earlier time periods for the whole United States.

For each point in time (i.e., every 5-years), we merged the resulting county-level raster datasets to state-wide surfaces, and finally to U.S.-wide layers (e.g., BUI_1865 for the year 1865) representing more than 2,600 out of 3,100 counties with good spatial and temporal coverage (see section C for more detail). In order to reduce the effect of incorrect built-up intensity values at boundaries between adjacent counties and states due to overlapping grid cells, we implemented the merging process in a way that it iteratively added the raster layers pixel-wise to a national-scale baseline raster of a constant initial value of zero.

As a second main data product, we created a raster layer, ***First Built-Up Year (FBUY)*** (Data Citation 3), in which each raster cell is assigned the earliest built year recorded among all point features located within the cell extent. This is the year of earliest built-up development in a given pixel (Figs. 1 and 2a,b). FBUY represents a temporal composite created from binarized, time-sliced BUI layers (built-up presence; Fig. 1) and can be used to reproduce such multi-temporal simple binary raster data layers of built-up vs. non-built-up land.

### Quality metrics and uncertainty surfaces as accompanying information layers

There are several important aspects of data quality that need to be addressed. We created different uncertainty surfaces at the county and pixel level (see Figs. 1 and 2) as accompanying data layers to evaluate the veracity and completeness of the main data products. We included these layers in our production effort to ensure potential users have immediate access to spatial, temporal and thematic uncertainty information for consultation and critical analysis when applying the final settlement data products in their research. We created the raster layers representing the pixel-level uncertainty measures to be consistent with the main raster data products, BUI and FBUY. We included the county-level uncertainty measures in the attribute table of a county-area vector file (***Cnty_Unc***, Data Citation 4). In the following section we describe the creation of these uncertainty layers.

### Spatial uncertainty

There are two aspects of spatial uncertainty in the database: the incompleteness in the coverage of geographic information and positional uncertainty of locations provided in the ZTRAX database.

#### County-level measures of completeness of geographic coordinates

Some counties have a proportion of records without geographic coordinates, and may contain records with duplicate coordinates resulting from spatial over-generalization or from coordinate imputation to substitute missing locational information (dummy records). For each county, we calculated the number of missing or invalid geographic coordinates and added this number to the attribute table of the county area vector file (**GeoMiss**) to be able to map this measure as a county-level variable (Fig. 2h and Data Citation 4) and calculate the proportion of missing values in relation to the total number of existing records (NumRecords) in the county.

#### Positional uncertainty at the pixel level

The geographic coordinates provided in the ZTRAX database are the result of an interpolation procedure and can be considered an approximation of address locations repositioned into their respective parcel unit. Thus, each record is subject to positional uncertainty that propagates through the aggregation process into the created raster layers and can affect the built-up intensity and first-built-up-year layers, differently. Therefore, this positional uncertainty has to be quantified and communicated to the data user. We created three different positional uncertainty raster layers for each of the main data products, i.e., the BUI and FBUY layers (Figs. 1 and 2d,e) with consistent raster properties. Each cell obtains an uncertainty value between 0 (certain) and 1 (completely uncertain).

The first uncertainty measure, the ***inside positional uncertainty IPU_BUI*** (Data Citation 2), is related to the positional uncertainty of all point features that are located inside a grid cell extent, but could potentially be located outside (uncertainty by commission error). For each of these point locations, we calculated the distance to the four cell boundaries. We derived the grid cell positions from the origin and cell size of the underlying template grid, which we could do efficiently after reprojecting the point records into the spatial reference system of the target grid. If a data record included a parcel area attribute, we calculated a circular buffer, describing the circumcircle of a square of the parcel area, centered at the given location (Fig. 2i). The square served as an approximation of the shape of the parcel unit. If this circle was completely inside the grid cell extent, this record obtains a weight of IPU_BUI=0 (certain). All other point features located inside the grid cell that either had a parcel area attribute but were located too close to a cell boundary (i.e., the circle overlapped with the cell boundary) or had no parcel area attribute at all were subject to uncertainty computation which we carried out as follows. For each of those points *i* we derived the distance to the closest cell boundary (dist_min,i_) and calculated IPU_BUI_i_ as:

$$IPU\;{}_{-}BUI_{i}=1-\frac{dist_{\min,i}}{0.5*\mathrm{res}}$$

The term 0.5*res is half of the cell dimension equal to 125 m, which is the maximum distance a point could be away from any cell boundary. Then, we summed up the IPU measures of all points inside the cell, and divided them by the number of inside points *n* to compute the average IPU_BUI, IPU_BUI_avg_, as:

$$IPU\;{}_{-}BUI_{avg}=\frac{1}{n}\sum_{i=0}^{n}IPU\;{}_{-}BUI_{i}=\frac{1}{n}\sum_{i-0}^{n}\left(1-\frac{dist_{\min,i}}{0.5*\mathrm{res}}\right)$$

For example, assume, a cell contains three points (Fig. 2i). One has a parcel area attribute for which the calculated buffer is completely inside the grid cell. The second one has a valid parcel area attribute but the buffer overlaps with an adjacent cell and is 70 m away from its closest cell boundary (Fig. 2i). The third point has no valid parcel area attribute and is 85 m away from its closest cell boundary. The average inside positional uncertainty, IPU_BUI_avg_, for this grid cell is calculated as IPU_BUI_avg_=(0+0.44+0.32)/3=0.2533.

The second measure we calculated is the ***outside positional uncertainty (OPU_BUI)*** (Data Citation 2), which plays an important role, as for each grid cell, there is a number of points that are located outside in neighbouring grid cells but rather close to one of its boundaries such that they could also be located inside (uncertainty by omission error). For each of these outside point locations, we calculated directional IPU_BUI measures, IPU_BUI_dir_, for each of the eight main directions, i.e., to the four cell boundaries where:

$$IPU\;{}_{-}BUI_{N,W,S,E,i}=1-\frac{dist_{N,W,S,E,i}}{\mathrm{res}}$$

and to the four cell corners, where

$$IPU\;{}_{-}BUI_{\text{NW},\text{NE},\text{SW},\text{SE},j}=1-\frac{dist_{\text{NW},\text{NE},\text{SW},\text{SE},j}}{\mathrm{res}*\sqrt{2}}$$

Next, for each grid cell, we identified the respective IPU_dir_ values found in the eight adjacent grid cells and calculated their average value to determine OPU as follows:.

$$OPU\;{}_{-}BUI_{\;{}_{-}avg}=\frac{1}{n+m}\left(\sum_{i=0}^{n}IPU\;{}_{-}BUI_{N,W,S,E,i}+{\sum_{j=0}^{m}IPU\;{}_{-}BUI}_{\text{NW},\text{NE},\text{SW},\text{SE},j}\right)$$

For example, for the pixel shown in Fig. 2i, there is one point in the adjacent cell to the East that has an IPU_BUI_W_ = 0.72 (35 m away from the cell boundary). In addition, there are two points in the northern neighbour with IPU_BUI_S_=0.4 and IPU_BUI_S_=0.96 (75 m and 5 m away from the cell boundary, respectively). These three uncertainty values are input to calculating OPU_BUI of the central grid cell as (0.72+0.4+0.96)/3=0.69.

Finally, we combined both uncertainty surfaces IPU_BUI and OPU_BUI to derive for each grid cell a measure of ***overall potential positional uncertainty (OPPU_BUI)*** (Data Citation 2) calculated as *OPPU_BUI=(IPU_BUI+OPU_BUI)/2*. As can be seen in the mathematical structure of these uncertainty calculations, a cell will receive a very low OPPU_BUI value if both IPU_BUI and OPU_BUI are low. IPU_BUI is low if all points inside are either close to the cell center or have very small parcel areas that are highly likely to be completely inside the grid cell. OPU_BUI is low if there are either no point features in neighbouring grid cells close to that cell’s boundaries or those close to the boundary have small parcel areas that are completely inside that neighbouring cell extent. In the example, outlined above (Fig. 2i), the overall uncertainty is OPPU_BUI = (0.2533+0.69)/2=0.472. We created these three uncertainty layers, IPU_BUI, OPU_BUI and OPPU_BUI, for each temporal snapshot of BUI (i.e., every 5 years) and added them as accompanying information layers. We urge the user to consult these layers in evaluating any analysis using the BUI raster layers. These values are relative uncertainty measures that have to be understood as “levels” of potential positional uncertainty between extreme cases.

For the FBUY raster layer (Data Citation 3), we calculate the positional uncertainty measures the same way but with a slightly modified set of input data. Similarly, we constrained positional uncertainty calculations for each cell to the structures within the cell boundaries and its neighbors that existed at the time indicated by the cell value. But we used only one of multiple point features that are encoded with the same geographic coordinates and for the same address, which is an indication of a multi-unit or even multi-story building with multiple units inside owned by different individuals or companies. To do this, we previously dissolved such multiple point features that would exist at a given point in time. This is opposed to the BUI positional uncertainty calculation, where all point features that included a valid building area attribute were taken into account, regardless the coordinate uniqueness. We produced the resulting positional uncertainty measures as raster composites with the advantage that only one layer per positional uncertainty type was necessary. We call these three raster composite layers ***OPU_FBUY, IPU_FBUY and OPPU_FBUY***(Data Citation 3).

### Temporal uncertainty

The raw database has a certain proportion of records for which the built-year attribute is missing. We created two products to help the user assessing temporal incompleteness when working with the settlement data. First, we computed a ***county level count of missing built year records (TMiss)*** and added it to the attribute table of the county area vector file (Cnty_Unc; Data Citation 4). This count can be used to calculate the proportion of built-year entries missing among all records within a county (***NumRecords***) which is also provided (Fig. 1).

Furthermore, we created a raster layer ***TPixMiss*** (Data Citations 2 and Data Citations 3 and Fig. 2f), consistent with the settlement layers, whose cell values are assigned the ***number of missing built-year records located within that cell***. TPixMiss can be used to map either the presence of missing records, the absolute number of missing records or the proportion of missing built-year records in relation to all existing records in that cell. We identified instances of identical built-years, so-called dummy entries, which are used to impute missing temporal information by the data producers across some counties. Once identified, we included these dummies as missing built-years.

Finally, there is some uncertainty about the accuracy of the built-year attribute because a built-year may refer to the structure established in a parcel or a structure that has been built after the first one has been torn down. This source of uncertainty cannot be quantified easily as it is often inherited from the official cadastral data and depends on the practice of data collection and maintenance in a specific county.

### Thematic uncertainty

The settlement layers are based on all ZTRAX records that describe parcel units with some kind of built-up structure. Whereas we assumed a high data quality given that the records are based on cadastral or other public data sources, we also expected that some of these assignments might be incorrect. Furthermore, in some instances, the ZTRAX database may indicate structures that do not exist (commission errors) or existing structures may not be registered in the database (omission errors). In the described data production effort we incorporated all records with a land use category that implies the existence of any kind of built structure. In the technical validation below we compared these records to original county-level parcel and building records to be able to characterize this classification uncertainty. However, we assessed the number of records within a county without an indication of land use (**LUMiss**) and added it as thematic uncertainty to the vector county area file (Data Citation 2).

We assessed a second component of thematic uncertainty, which is introduced by potentially missing building area information. This can result in an underestimation of the gross building area in the resulting BUI layers and thus represents relevant accompanying information. In order to account for this, we identified all point features in each time slice that do not have a valid gross building area attribute. We then used these selected features to create a series of raster layers, **APixMiss** (Data Citation 2 and Fig. 2g) in which we assigned each cell value the number of these missing records within the cell extent for each point in time. This allows the user to assess the completeness of built-up intensities in the BUI layers at the pixel-level and for each point in time. We also calculated a county level measure of these counts over all points in time and included it in the attribute table as **AMiss** (Data Citation 4) as a more generalized aggregated uncertainty measure for data users.

### Code availability

We wrote the source code for data processing in Python and SQL, including the SQLite database queries, the creation of vector (point) files, the calculation of the settlement measures and uncertainty measures as described above, and the rasterization of the point features to create the settlement and uncertainty layers. We also created Python scripts for carrying out the validation tests described in this descriptor. Our scripts can be obtained per request.

## Data Records

### Input: ZTRAX database

Zillow’s ZTRAX database contains unique data on housing transactions, home values, rental estimates, spatial location, home- and property-related information as well as built-year information, for existing homes and certain other properties across the United States (Table 1). As reported by the owner of the data, Zillow Group, the database includes information from more than 374 million public records and assessor data for approximately 200 million parcels in over 3,100 counties in the U.S. (http://www.zillow.com/ztrax). The data are obtained from a major large third-party provider as well as through an internal initiative, called County Direct. County Direct prioritizes counties based on different characteristics and supplements the third-party coverage by collecting data directly from county Assessor and Recorder’s offices, and represents a growing share of the ZTRAX dataset. These data provide unique opportunities for historical analysis of residential land use, housing markets and the built structure. Foremost of interest in this study, we assess micro-scale measurement of the evolution of residential and non-residential land use related to built structures using land use information, building area and construction year information. Furthermore, the ZTRAX database is a rich source of geospatial data in the form of approximate address point locations and address information enabling the characterization of settlement activity at fine spatial and temporal resolution (Fig. 2).

While this database is of unique nature covering most of the U.S. there are some issues related to data quality as described above, including spatial, temporal and thematic uncertainties that we assessed and measured in order to create accompanying uncertainty layers. The database is under continuous revision and will be updated regularly. As explained before, the raw data and the created SQLite databases must not be shared publicly per the established data share agreement.

### Output: Historical settlement raster layers

The settlement raster layers can be found online (Fig. 2 and Table 1). The **built-up intensity**, ***BUI*** (Data Citation 2) is a raster time series (file names include the year e.g., BUI_1865), with a temporal resolution of 5 years. Each raster cell value represents the sum of the gross indoor area (square meters) of all structures that are located inside the cell extents in a given year or before (Fig. 2).

The **First Built-Up Year*****, FBUY*** (Data Citation 3) is a single raster composite with a temporal resolution of five years. The FBUY cell values represent the year in which the first structure has been established in that raster cell, at a temporal granularity of five years.

Both data products cover the time period 1810–2015. We provide the raster layers in Geo-TIFF format with a spatial resolution of 250 m. We aligned these layers to the GHSL 250 m built-up data grid in order to ensure compatibility with these remote-sensing derived built-up land layers between 1975 and 2014 (ref. 13). The datasets are published in North America Albers Equal Area Conic Projection (EPSG 102008).

### Output: Accompanying uncertainty surfaces

As described above, we created several uncertainty surfaces in order to provide information on basic data quality aspects (Fig. 2 and Table 1). We produced and provided one set of uncertainty surfaces as raster data at the same resolution as the settlement layers. This includes the three time series of positional uncertainty layers (***IPU_BUI, OPU_BUI and OPPU_BUI***) (Data Citation 2), and a time series of building area missingness counts (***APixMiss***) (Data Citation 2), all accompanying the BUI layers. We also produced the three raster composite uncertainty surfaces accompanying the FBUY raster composite (***IPU_FBUY, OPU_FBUY and OPPU_FBUY***) (Data Citation 3). And finally, we created the temporal uncertainty raster layer, the number of missing built-year records located within a grid cell (***TPixMiss***) (Data Citation 2 and Data Citation 3).

The second category of uncertainties we produced includes different county-level incompleteness measures all of which we added to the attribute table of the county area vector file (***Cnty_Unc***) (Data Citation 4), as described above (Fig. 2 and Table 1). These are counts of missing records for different relevant attributes including built-year information (***TMiss***), geolocation (***GeoMiss***), land use class (***LUMiss***), and building area (***AMiss***), all of which can be used to calculate county level proportions of missing records in relation to the total number of records in these counties which is delivered as well (***NumRecords***). We produced these raster layers and county level measures of incompleteness in order to ensure the user community is able to use the settlement layers in reflected and critical ways.

### Validation data: Temporally encoded fine resolution building footprints

For some administrative regions in the U.S. (29 counties) in which parcel data with the year a structure has been built are available, we could also access building data to create a unique spatio-temporal database covering different geographic settings in the U.S.

Open cadastral and tax assessment (parcel) data have become increasingly available to the public–often in GIS-compatible format – for several regions in the U.S.^23^ and other countries. For this study, we used all publicly available parcel data that we obtained through open-sources. Cadastral parcel boundaries are typically acquired using terrestrial or satellite technology-based land surveying methods. Parcel data usually contain rich attribute information related to the type of land use, characteristics of the structures and the year when a structure in a parcel has been established (built year). This allows the creation of snapshots of built-up parceled land for any point in time with a temporal resolution of one year. While a valuable data source for modeling areas of residential development^24^ and population estimates for small areas^25^ and different points in time^26^, the use of parcel data can be challenging. Such data are still difficult to obtain for most areas and can suffer from attribute inconsistencies^27^. Furthermore, there is some significant size variation in land parcel units, especially across urban-rural trajectories, which needs to be taken into account if parcels are used as analytical units^28^.

Data representing building footprints are typically derived from LiDAR measurements or digitized based on aerial imagery. With increasing investments in detecting and mapping existing structures, building data are becoming increasingly available as open data. We used such open datasets in this study to spatially refine the snapshots of built-up parcelled land. This refinement is expected to be especially effective in rural areas where parcel units can have large areal extents and are expected to drastically overestimate built-up land if they remain unrefined.

This validation dataset has previously been applied to quantify the accuracy of multi-temporal built-up land layers (namely the Global Human Settlement Layer, GHSL)^14^. It covers different regions of the U.S. and thus different landscape conditions, vegetation types and settlement histories. In total, we collected data for 29 counties summing to an area of >40,000 km^2^, and containing almost 6 million parcel features across the U.S. These 29 counties show great variation in areal extent and urban character as well as in the rate of development over time. While it can be assumed that these official cadastral and building data have high quality standards, detailed accuracy information is not available for most of these datasets. There are several differences between the ZTRAX data and these integrated validation data due to data processing and harmonization processes conducted by Zillow and third party data providers.

## Technical Validation

For the two main data products, the BUI raster time series and the FBUY composite raster layer, we conducted a thorough validation using the above described validation dataset. We evaluated FBUY in a multi-class accuracy assessment and BUI in a regression framework as described below. We tested for temporal and spatial aggregation effects to expose the potential sensitivity of the accuracy to those effects and to provide validation results for different use scenarios.

### Assessing the accuracy of the first built-up year raster composite layer, FBUY

In order to assess the accuracy of FBUY we converted the reference dataset into a raster layer consistent with FBUY and assigned the first built up year found in the reference data (***FBUY***_***ref***_) within a raster cell as the raster value. We carried out a multiclass evaluation by pixel-wise comparison of FBUY with FBUY_ref_ over all 29 reference counties, and for three levels of spatial aggregation i.e., at resolutions 250 m, 750 m and 1,250 m. We also tested two levels of temporal aggregation by discretizing FBUY and FBUY_ref_ into semi-decadal and decadal time steps.

Additionally, we used the time series of binary built-up layers for each point in time, which we created in an intermediate step during FBUY production as described above, and compared them to the binary form of cumulative built-up layers created from the validation data for each point in time. Based on these comparisons we created a two-class confusion matrix for each point in time and computed a series of different accuracy measures, which we then plotted over time. We conducted this accuracy assessment for the three levels of spatial aggregation mentioned above.

Based on the time series of two-class confusion matrices established at different spatial aggregation levels, we computed various accuracy measures including traditional ones such as percent correctly classified (PCC), Kappa coefficient of agreement (K)^29^, or precision and recall, but also measures that have been developed explicitly to address potentially unbalanced data distributions such as the Normalized Mutual Information criterion (NMI)^30^, the geometric mean (G-mean), which takes into account sensitivity and specificity^31^ and the F-measure, the harmonic mean of recall and precision^32^. The issue of unbalanced distributions represents an important aspect to derive objective assessment results, because we expected that the distribution of built-up land would be highly biased i.e., high proportions of built-up, low proportions of non-built-up in urban areas, and vice versa in rural regions.

**Figure 3:**
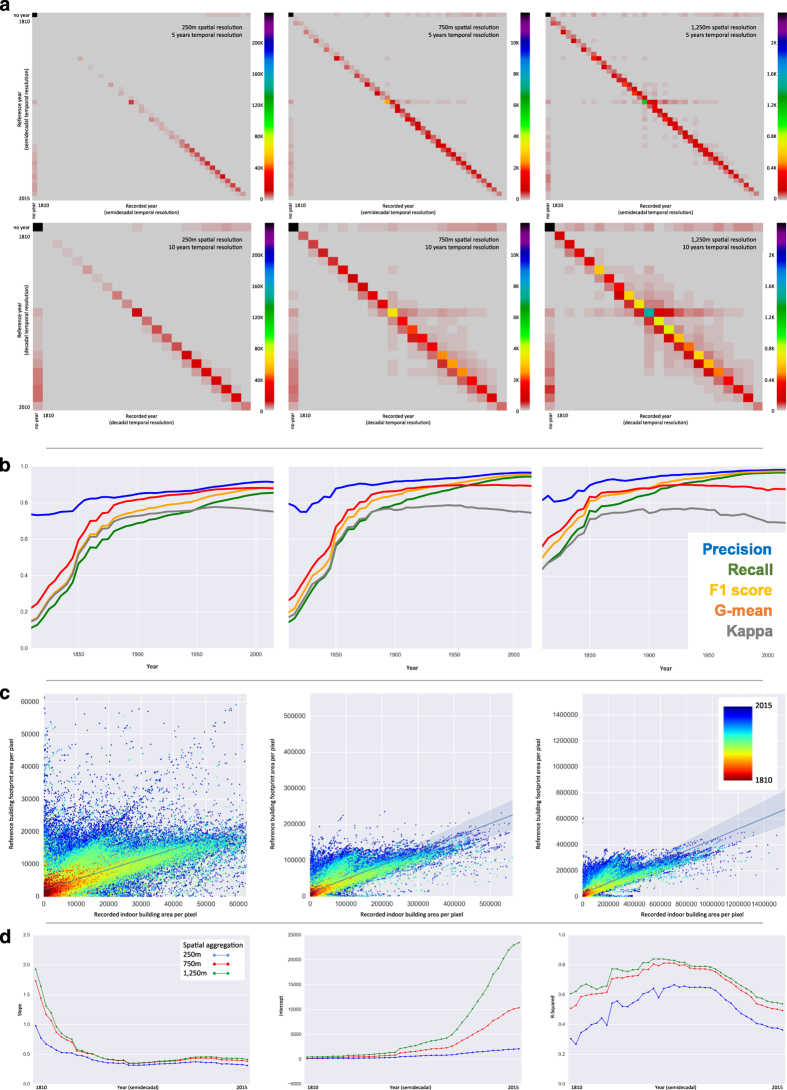
Results from the evaluation experiments for FBUY and BUI using the reference data. (**a**) Confusion matrices for the FBUY classes at 5-year (upper row) and 10-year (lower row) temporal aggregation, each for spatial aggregations of 250 m, 750 m, and 1,250 m (from left to right). (**b**) Accuracy measures (precision, recall, G-mean, F-measure and Kappa) of semi-decadal FBUY layers plotted over time, for spatial aggregations of 250 m, 750 m, and 1,250 m (from left to right). (**c**) Scatterplots to show the association between BUI and BUI_ref_ values, color-coded for different time periods, based on spatial aggregations of 250 m, 750 m, and 1,250 m (from left to right); regression lines shown are estimated for the regression of BUI to estimate BUI_ref_ in 2015. (**d**) Slope, intercept and R-squared (from left to right) for linear regression analyses of BUI to estimate BUI_ref_ for all points in time between 1810 and 2015, at spatial aggregations of 250 m (blue), 750 m (red), and 1,250 m (green).

The multi-class confusion matrices in Fig. 3a illustrate a trend of increasing agreement between test and reference data with increasing spatial and temporal aggregation. This was likely due to the fact that existing spatial and temporal offsets between the two datasets could be mitigated to some part, elevating the general agreement in individual points in time along the diagonal of the multi-class confusion matrices. The spatial aggregation effect is also illustrated by the plotted accuracy measures over time but even at the finest aggregation level we found acceptable levels of agreement for most accuracy measures from 1850 onward (Fig. 3b). In some counties, we identified issues related to dummy built-years for earlier time periods resulting in relatively low measures of agreement (less than 0.4 for recall, F-measure and Kappa). However, even for these very early time periods, we found high precision indicating very low rates of false positives. Overall, all accuracy measures are close to or greater than 0.7 after 1875 even at 250 m resolution giving us high levels of confidence in the FBUY composite and the binary settlement layers that can be created for each point in time.

### Evaluating the built-up intensity layers, BUI

As described above, we created the BUI raster layers based on the gross indoor building area provided by Zillow. Our reference data do not include the same variable, consistently. Thus, we used the measured building footprint area as the reference measure and evaluated how well each BUI layer can predict this reference measure in a regression framework. While these measures are not perfectly compatible, this evaluation would still allow us to examine an important association for which we expect certain trends over time. For example, we expected that BUI would measure the building footprint area, reliably, in early time periods, but overestimate the reference measure in more recent years with the increased construction of multi-story buildings.

Using the reference data, we created a series of gridded layers, ***BUI***_***ref***_, consistent with the BUI layers, using the cumulative building footprint areas of buildings within a raster cell as raster values for each point in time. We created three sets of BUI_ref_ raster layer series, one for each spatial aggregation level (i.e., 250 m, 750 m, 1,250 m). We then conducted a regression analysis based on pairs of spatially coinciding pixel values of BUI and BUI_ref_, separately, for each point in time, resulting in time series of linear regression models for the three different aggregation levels. We explored the variation of slopes, intercepts, and R-squared measures of the regression models over time to better understand this relationship and its changes.

The scatterplots in Fig. 3c, which include the regression lines for the 2015 epoch, illustrate a strong relationship between both variables for earlier time periods, but indicate a systematic overestimation of BUI_ref_ in recent years (in blue) as expected. The slope of the regression line confirms this overestimation effect in 2015 mainly caused by the differences between indoor area of a building and the area of the building footprint for multi-story buildings. However, we also identified strong linear relationships of slope close to 1 for single-floor buildings. As shown in Fig. 3d, we could observe a decrease in the regression slopes until 1900, which stabilized after that around 0.4, when high-rise buildings were increasingly built. Accordingly, the R-square values increase between 1810 and 1900 and decrease thereafter.

As expected, the relationship between BUI and BUI_ref_ becomes more robust for coarser spatial resolutions but shows the same trends as described above. Our tests are of confirmatory nature and provide important insights for evaluation of the BUI layers in addition to the FBUY validation.

## Usage Notes

We organized the data in the Harvard Dataverse repository (https://dataverse.harvard.edu/) by creating a dataverse “***HISDAC-US: Historical Settlement Data Compilation for the United States***” (https://dataverse.harvard.edu/dataverse/hisdacus). We added three different datasets to this dataverse each of which contains one or more compressed archives. In each dataset the user can find a readme.txt file, the data products and uncertainty layers organized in a file directory system as described below:

Dataset 1: Historical built-up intensity layer series for the U.S. 1810 – 2015 (Data Citation 2):

- ***BUI.tar.gz***: Archive contains the time series of BUI raster layers between 1810 and 2015 (BUI_1810 … BUI_2015) with semi-decadal temporal resolution.
- ***BUI_IPU.tar.gz:*** Archive contains IPU raster time series [1810–2015] accompanying BUI
- ***BUI_OPU.tar.gz:*** Archive contains OPU raster time series [1810–2015] accompanying BUI
- ***BUI_OPPU.tar.gz:*** Archive contains OPPU raster time series [1810–2015] accompanying BUI
- ***TPixMiss.tar.gz:*** Archive contains the raster layer representing the number of missing built year records per pixel
- ***APixMiss.tar.gz:*** Archive contains the raster time series [1810–2015] representing the number of missing indoor area records per pixel

Dataset 2: Historical settlement composite layer for the U.S. 1810–2015 (Data Citation 3):

- ***FBUY.tar.gz:*** Archive that contains the FBUY raster composite (a value of 1 indicates the presence of built structures without built-year record only)
- ***FBUY_IPU.tar.gz:*** Contains the IPU raster layer accompanying FBUY
- ***FBUY_OPU.tar.gz:*** Contains the OPU raster layer accompanying FBUY
- ***FBUY_OPPU.tar.gz:*** Contains the OPPU raster layer accompanying FBUY

Dataset 3: County-level uncertainty statistics accompanying the historical settlement layers for the U.S. 1810–2015 (Data Citation 4)

- ***County_level_uncertainty.tar.gz:*** Uncertainty measures at the county level:

***Cnty_unc***: County-boundary vector layer Cnty_Unc.shp with several county level uncertainty measures in the attribute table Cnty_Unc.dbf (Table 1); the names of the fields are:

*TMiss*: Records with missing built-year per county (temporal)

*GeoMiss*: Records with missing geographic coordinates per county (spatial)

*LUMiss*: Records with missing land use attribute per county (thematic)

*AMiss*: Records with missing building area per county (thematic)

*NumRecords* is an additional field that presents the total number of records in a county and can be used to calculate proportions of missing records.

We ask the reader to refer to Table 1 and the descriptor text for detailed explanations of the different data layers. Each tar.gz archive above contains a readme.txt file for documentation of the contents and the data structure. Furthermore, all tar.gz archives above contain symbology files for QGIS (.qlr) and for ArcGIS (.lyr) for convenient display of the settlement data and the uncertainty data using predefined symbology.

We highly recommend that users who apply our settlement data products use them in combination with the provided uncertainty surfaces. This will help the data user to make correct and objective interpretations in their analysis under the consideration of inherent uncertainty. The different data products have been linked to Digital Object Identifiers (DOIs). These DOIs will be used to link different web presences of relevant projects of the researchers and Zillow Research to the Harvard Dataverse. Furthermore, the data will be hosted at servers maintained by the University of Colorado.

## Additional information

**How to cite this article**: Leyk, S. & Uhl, J. H. HISDAC-US, historical settlement data compilation for the conterminous United States over 200 years. *Sci. Data* 5:180175 doi: 10.1038/sdata.2018.175 (2018).

**Publisher’s note**: Springer Nature remains neutral with regard to jurisdictional claims in published maps and institutional affiliations.

## Supplementary Material



## Figures and Tables

**Table 1:** Descriptions and characteristics of input, output and reference data used in this study.

Name/Category	Short Description	Data type	Temporal resolution	Spatial resolution
***Input***				
*ZTRAX database*	Housing and property database provided by Zillow, with geolocation and temporal information	CSV	annual	point locations
***Output - main data products***				
*Built-up intensity (BUI)*	Sum of gross indoor area (in m^2^) of all structures located within a given cell at a given year (cumulative) 1810-2015	Geo-TIFF, raster series, Integer	Semi-decadal	250 m
*First Built-Up Year (FBUY)*	First recorded year a structure has been built within a given cell extent; first year of settlement	Geo-TIFF, raster composite, Integer	Semi-decadal	250 m
***Output - uncertainty layers***				
*Inside Positional Uncertainty (IPU_BUI)*	For each cell in BUI rasters, the uncertainty that a point located inside could be outside; accompanying BUI raster series	Geo-TIFF, raster series, Float	Semi-decadal	250 m
*Outside Positional Uncertainty (OPU_BUI)*	For each cell in BUI rasters, the uncertainty that a point located in adjacent cells could be inside; accompanying BUI raster series	Geo-TIFF, raster series, Float	Semi-decadal	250 m
*Overall Potential Positional Uncertainty (OPPU_BUI)*	For each cell in BUI rasters, the average positional uncertainty, accompanying BUI raster series	Geo-TIFF, raster series, Float	Semi-decadal	250 m
*Inside Positional Uncertainty (IPU_FBUY)*	For each cell in FBUY, the uncertainty that a point located inside could be outside, limited to unique locations of structures recorded at the time; accompanying FBUY raster composite	Geo-TIFF, raster composite, Float	Semi-decadal	250 m
*Outside Positional Uncertainty (OPU_FBUY)*	For each cell in FBUY, the uncertainty that a point located in adjacent cell could be inside, limited to unique locations of structures recorded at the time; accompanying FBUY raster composite	Geo-TIFF, raster composite, Float	Semi-decadal	250 m
*Overall Potential Positional Uncert. (OPPU_FBUY)*	For each cell in FBUY, the average positional uncertainty, limited to unique locations of structures recorded at the time; accompanying FBUY raster composite	Geo-TIFF, raster composite, Float	Semi-decadal	250 m
*Temporal Uncertainty (TPixMiss)*	For each cell, the number of missing built-year records; accompanying FBUY raster composite	Geo-TIFF, raster layer, Integer	N/A	250 m
*Thematic Uncertainty (APixMiss)*	For each cell, the number of missing records for building indoor area for each point in time; accompanying BUI raster series	Geo-TIFF, raster series, Integer	Semi-decadal	250 m
*County-level (Cnty_Unc) uncertainty:*	Vector data, delineating county boundaries in 2010 with various uncertainty measures in the attribute table for mapping uncertainty `	ESRI shapefile	N/A	County
-> TMiss:	The sum of missing built year records in a county	Attribute, Integer	N/A	County
-> GeoMiss:	The sum of missing or invalid geographic coordinates in a county	Attribute, Integer	N/A	County
-> LUMiss:	The sum of records without landuse in a county	Attribute, Integer	N/A	County
-> AMiss:	The sum of missing records for building indoor area over all years in a county	Attribute, Integer	N/A	County
***Reference layers based on integrated building footprints***				
Reference-Built-up intensity (BUI_Ref_)	For each cell, the sum of building footprint area (in m^2^) of all structures located inside at a given year (cumulative) 1810-2015	Geo-TIFF, raster series, Integer	Semi-decadal	250 m
Reference-First Built-Up Year (FBUY_Ref_)	For each cell, the earliest built-up year reported used for validation of FBUY	Geo-TIFF, raster composite, Integer	Semi-decadal	250 m
